# The safety and immunogenicity of a MF59-adjuvanted H5N1 prepandemic influenza vaccine in healthy adults primed with homologous or heterologous H5N1 vaccines: an observational study

**DOI:** 10.1186/s12879-014-0587-z

**Published:** 2014-11-14

**Authors:** Sung-Hsi Wei, Ming-Tsan Liu, Yao-Chou Tsai, Chung-Hsin Liao, Chih-Ming Chen, Wei-Yao Wang, Yi-Lung Huang, Feng-Yee Chang, Pesus Chou

**Affiliations:** Community Medicine Research Center and Institute of Public Health, National Yang-Ming University, No 155, Sec 2, Linong St., Beitou Dist, Taipei City, 112 Taiwan; Centers for Disease Control, No.6, Linsen S Rd, Zhongzheng Dist, Taipei City, 10050 Taiwan; Department of Internal Medicine, Changhua Hospital, Ministry of Health and Welfare, No 80, Sec 2, Zhongzheng Rd, Puxin Township, Changhua County, 513 Taiwan; Department of Internal Medicine, Tungs’ Taichung MetroHarbor Hospital, No 699, Sec 8, Taiwan Blvd, Wuqi Dist, Taichung City, 435 Taiwan; Department of Internal Medicine, Feng Yuan Hospital, Ministry of Health and Welfare, No.100, Ankang Rd., Fengyuan Dist, Taichung City, 420 Taiwan; Division of Infectious Diseases and Tropical Medicine, Department of Internal Medicine, Tri-Service General Hospital, National Defense Medical Center, No. 325, Sec. 2, Cheng-Kung Rd, Neihu, Taipei City, 114 Taiwan

**Keywords:** A/Vietnam/1194/2004, A/Indonesia/05/2005, H5N1 vaccine, Priming

## Abstract

**Background:**

World Health Organization (WHO) has recommended individuals with increased risk of contracting influenza A H5N1 infection to be immunized against the virus during the inter-pandemic period. Safety and immunogenicity of H5N1 vaccine among participants primed with homologous or heterologous H5N1 vaccines produced by diverse manufactures have not been reported.

**Methods:**

Healthy individuals aged 20 to 60 years old were recruited and stratified into three groups: participants without priming (control group), participants primed with A/Indonesia/05/2005 vaccine, participants primed with A/Vietnam/1194/2004 vaccine and A/Indonesia/05/2005 vaccine. Enrolled participants received two doses of MF59-adjuvanted A/Vietnam/1194/2004 vaccine (study vaccine). Solicited reactions were recorded by vaccine recipients. Blood samples were obtained for hemagglutination inhibition test.

**Results:**

A total of 131 participants were enrolled. No significant adverse events were recorded. Tenderness, fatigue and general muscle ache were the most common solicited reactions which alleviated within one week of immunization. Three weeks after two doses of the study vaccine, 63%, 68% and 88% were in seroprotective status in the control group, A/Indonesia/05/2005 primed group and A/Vietnam/1194/2004 and A/Indonesia/05/2005 primed group, respectively. Participants primed with A/Vietnam/1194/2004 and A/Indonesia/05/2005 showed high immune response after booster with one dose of the study vaccine.

**Conclusion:**

The study vaccine did not cause severe adverse events. It elicited mostly mild to moderate reactions among participants. Participants primed with A/Vietnam/1194/2004 and A/Indonesia/05/2005 vaccine showed higher immune response than those without priming or primed with A/Indonesia/05/2005 vaccine. The report suggested those with an increased risk of influenza A H5N1 virus exposure may benefit from receiving influenza A H5N1 priming during the inter-pandemic period if the antigenicity of the pandemic influenza strain is similar to that of the priming strain.

**Electronic supplementary material:**

The online version of this article (doi:10.1186/s12879-014-0587-z) contains supplementary material, which is available to authorized users.

## Background

Avian influenza A H5N1 virus was first identified to infect human in Hong Kong in 1997, killing 6 of 18 infected persons [1]. Since then, influenza A H5N1 infection was largely confined in Southeast Asia until 2006 when patients in Turkey contracted the virus [2]. As of Dec 20, 2013, influenza A H5N1 virus had caused 648 cases of human infection; among them, 384 (59.3%) died [3]. The significant morbidity and mortality outcomes caused by influenza A H5N1 infection pose a major threat for the next global pandemic.

With the genetic evolution of more than 15 years, influenza A H5N1 virus has evolved to clade 1 and clade 2; the latter could be further divided into subclades [1]. Significance of the clade classification is not only on the susceptibility of antiviral agents but also on the antigenicity, which warrant the preparation of different kinds of H5N1 vaccines [4].

Influenza vaccination is one of the cornerstones of pandemic influenza preparedness. Strategic Advisory Group of Experts (SAGE) of WHO has recommended individuals with increased risk of influenza A H5N1 exposure to receive influenza A H5N1 immunization during the inter-pandemic period, e.g., laboratory workers involved in certain risk activities, first responders to human or animal high pathogenic avian influenza (HPAI) H5N1 cases or outbreaks, health-care workers who evaluate or manage patients with suspected or confirmed HPAI H5N1 virus infection in designated referral facilities [5]. Although there have been licensed H5N1 vaccines for use in the inter-pandemic period, information on the use of the vaccines remains limited. It is encouraged to gain experience on the safety, immunogenicity, cross-reactivity, priming potential of the H5N1 vaccines and duration of the elicited immunity [6].

Taiwan has developed integrated programs and dedicated huge resources to the pandemic preparedness in the past decade, including stockpiling of prepandemic vaccines of clade 1 and clade 2 H5N1 viruses [7],[8]. Taiwan government had provided H5N1 vaccine to personnel with risk of H5N1 virus exposure on 2008, 2010 and 2011. Taiwan Advisory Committee on Immunization Practices (TACIP) evaluated the pandemic threat and recommended the subjects eligible for influenza A H5N1 immunization in each of the abovementioned years. Individuals at risk of H5N1 virus exposure might be eligible for receiving influenza A H5N1 immunization for more than one time and received more than one course of influenza A H5N1 immunization in the past years. However, the experience of the influenza A H5N1 immunization in Taiwan is rarely reported [9]. Here we report the safety and immunogenicity profile of an influenza A H5N1 vaccine provided in 2011 for individuals with or without previous homologous or heterologous H5N1 priming.

## Method

### Settings and participants

TACIP recommended the following individuals 18 years of age or elder to be eligible for influenza A H5N1 immunization in 2011: health care workers, especially those who work in the assigned referral facilities [7], poultry workers including poultry farm workers and poultry slaughterhouse workers, agriculture officers in charge of poultry health, zoo workers, costal guardians, custom officers and citizens who are going to countries with documented avian influenza H5N1 infection. We provided the immunization service and recruited vaccine recipients for participating in the study. Healthy vaccine recipients aged 20 to 60 years were eligible and were consulted for participating in the study. Those who had underlying diseases, e.g., malignancies, diabetes, hypertension, were excluded from the enrollment. Other exclusion criteria include any allergy history to components of influenza vaccine or adverse events after influenza immunization, fever illness within seven days before the H5N1 immunization, pregnancies or intending to be pregnant.

**Figure 1:**
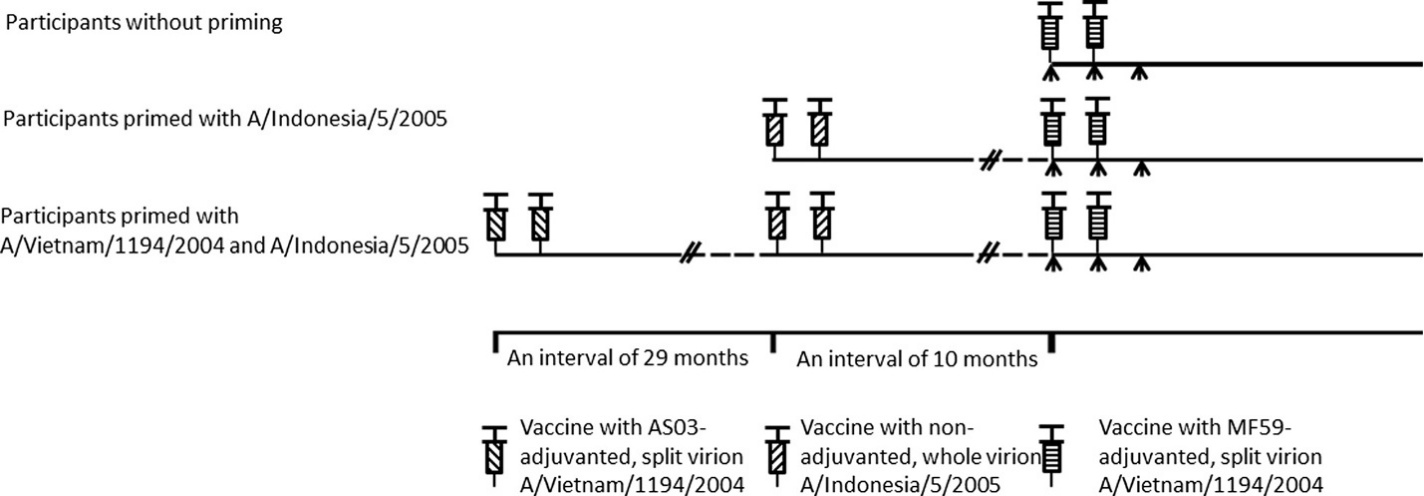
**Study design.** AS03-adjuvanted split virion A/Vietnam/1194/2004 vaccine was provided and primed in March and April, 2008. Non-adjuvanted whole virion A/Indonesia/05/2005 vaccine was provided and primed in August and September, 2010. The study vaccine of MF59-adjuvanted split virion A/Vietnam/1194/2004 virus was administered in Jun and July, 2011. Each course of vaccine administration included two doses of immunization apart by three weeks. Arrow indicates blood sampling for Hemagglutination inhibition test.

The influenza A H5N1 immunization records of the participants in 2008 and 2010 were obtained from Taiwan Centers for Disease Control (TCDC) which then provided the prepandemic influenza vaccine and maintained the records. Enrolled participants were stratified into three groups according to their previous influenza A H5N1 immunization records: participants without priming (control group), participants primed with A/Indonesia/05/2005 vaccine in 2010, and participants primed with A/Vietnam/1194/2004 vaccine in 2008 and A/Indonesia/05/2005 vaccine in 2010 (Figure 1). Those who had only received one dose of H5N1 vaccine in 2008 or 2010 were excluded from the analysis.

### H5N1 vaccine and priming

The study was performed in June and July, 2011. The H5N1 vaccine used in the study was a licensed prepandemic influenza A H5N1 vaccine, with the brand name of Aflunov, and was manufactured by Novartis Vaccines and Diagnostics (Siena, Italy) [10],[11]. It is an egg-derived, inactivated subunit influenza vaccine adjuvanted with MF59, an oil in water emulsion which in influenza vaccine has been shown to increase the immune response to influenza virus strains [12],[13]. Each dose of the H5N1 vaccine contains 7.5 μg of purified hemagglutinin of clade 1 influenza A H5N1 strain which is a reference strain derived by reverse genetics from a HPAI strain A/Vietnam/1194/2004 by the UK National Institute for Biological Standards and Control [14]. The vaccine is intramuscularly delivered for two doses apart by three weeks.

Previous priming immunization was administered in 2008 and 2010. For the former priming, two dose of egg based, inactivated split-virion influenza A/H5N1 vaccine, manufactured by GlaxoSmithKline (GSK) Biologicals (Dresden, Germany), were delivered with an interval of 3 weeks during March and April, 2008 [15]. The vaccine contains 3.75 μg of hemagglutinin antigen and is adjuvanted with AS03, an oil in water emulsion which augments the immune response [15],[16]. The vaccine seed virus, the H5N1 reassortant reference virus A/Vietnam/1194/2004, was the same as that in Aflunov administered in 2011 in the study. For the latter priming, two doses of cell-based, unadjuvanted, inactivated whole virion influenza A/H5N1 vaccine, manufactured by Baxter Pharmaceutics (Vienna, Austria), were delivered with an interval of 3 weeks in August and September, 2010. The vaccine contained 7.5 μg of wild type strain A/Indonesia/05/2005, which was a clade 2.1 influenza A H5N1 virus strain. No egg or chicken protein was contained in the vaccine [17].

### Adverse events monitoring

A questionnaire was developed to evaluate the local reactions (e.g., redness, swelling, tenderness, indurations, purpura around the injection sites) and systemic reactions (e.g., fever, chillness, headache, fatigue, muscle ache) within 7 days of immunization. Presence of the local or systemic reaction was determined according to the participants’ perception. Severe redness is defined as redness with a diameter of greater than 50 mm around the injection site. Severe swelling is defined as an extension in the circumference of vaccine-administered upper arm for greater than 30 mm than that of the counterpart upper arm, or swelling at the vaccine-administered upper arm with distal digits numbness. Severe tenderness is defined as significant pain at the vaccine-administered arm with which the participants failed to carry out daily activity and had to leave from work. Severe induration is defined as a subcutaneous induration with a diameter of greater than 50 mm. Severe fever is defined as an elevated body temperature of greater than 39.5 degree Celsius. Severe systemic reactions of chillness, headache, fatigue, or muscle ache were defined as significant discomfort due to the abovementioned symptoms with which the participants failed to carry out daily activity and had to leave from work. The questionnaire was sent to the participants with email in advance of the immunization. Physical questionnaire was delivered to the participants after each of the immunization was completed. The participants evaluated the reactions and completed the questionnaire themselves. They recorded the reactions electronically or by paper-pencil method, and returned the questionnaire in one to two weeks after each immunization.

### Laboratory method

For each participants, three set of blood samples were collected before the first dose of immunization, before the second dose of immunization, and three weeks after the second dose of immunization, respectively. All the blood samples were placed in the room temperature for 40 minutes; then the samples were centrifuged to obtain the serum samples in the study hospital or commercial medical laboratories. The serum samples were then sent to the laboratory in TCDC for hemagglutination inhibition (HI) test. Serial serum dilutions, started at 1:5, were tested for HI titers according to the method developed previously [18]. Since sialic acid of α2,3-galactose linkages on erythrocyte was the target of HI test against avian influenza, horse erythrocyte, which is abundant in this kind of sialic acid, was used in the test [19]. Influenza A/Vietnam/1194/2004 vaccine reference strain was used as test antigen to test for specific antibodies. HI titers were reported as the reciprocal of the highest dilution of serum that inhibited virus-induced hemagglutination completely. The HI test was measured with a maximal dilution of 1:640. Positive HI test with a dilution of greater than 1:640 was recorded as a HI titer of 640. Seroprotective status is defined as a HI titer of 40 or greater. Seroconversion rate is defined as the proportion of participants who have a reciprocal titer of less than 10 before vaccination and a titer of more than 40 after vaccination or have a HI titer of more than 10 before vaccination and at least a four-fold increase in titer after vaccination. All the laboratory staff were blind with the demographic and the priming status of the participants.

### Data analysis and statistics

We use Epi Info version 3.5.1 for data entry for the reactogenicity evaluation and laboratory results. The statistical analysis was completed using Stata 11 software. For categorical comparison, chi square test or Fisher’s exact test, in the circumstances of a value of 5 or less in any cell, were used. A p value of less than 0.05 was regarded as significant.

### Ethical consideration

The study protocol was approved by the institutional review board of TCDC (reference number, TwCDCIRB100020). Written informed consent was obtained from the enrolled participants after the nature and possible consequences of the studies had been fully explained.

## Results

**Figure 2:**
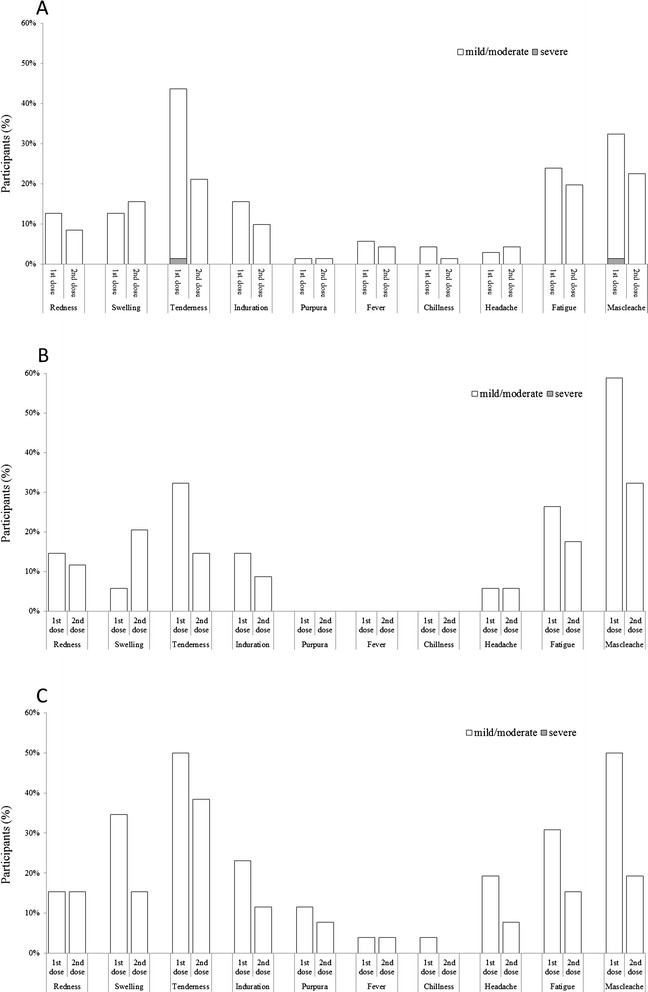
**Reported incidences of local and systemic reactions after first and second dose of immunization.** White bar indicates mild to moderate reaction while gray bar indicates severe reaction. **A**, **B** and **C** indicate the incidence of reaction among participants without priming, participant primed with A/Indonesia/05/2005 and participants primed with both A/Vietnam/1194/2004 and A/Indonesia/05/2005, respectively.

A total of 145 individuals participated in the study. One was excluded from the study because of difficulty in blood sampling. Seven participants who were only primed with two doses of A/Vietnam/1194/2004 vaccine in 2008 and six participants who had received only one dose of A/Vietnam/1194/2004 vaccine in 2008 were excluded from analysis. The demographic data of the remaining 131 participants is shown in Table 1. The rates of local and systemic solicited reaction were shown in Figure 2. During the study period, there was no immediate anaphylactic reaction. All the local and systemic solicited reaction alleviated within 7 days of vaccine administration and none was hospitalized for the solicited reactions. The most common local reactions were tenderness around the injection site (42% and 23% for the first and second dose of immunization, respectively). Generally, the rates of solicited local reaction were higher in participants with previous A/Vietnam/1194/2004 and A/Indonesia/05/2005 priming than those without priming or with previous A/Indonesia/05/2005 priming. However, no significant difference was found in the rates of solicited local reactions among the three groups, except for the swelling after first dose of immunization (p = 0.01). One participant without priming history developed severe injection site tenderness after first dose of immunization and left from work for one day because of the local reaction.

**Table 1:** **The demographic data of the enrolled participants**

	Participants without priming (n = 71)	Participants primed with A/Indonesia/05/2005 (n = 34)	Participants primed with A/Vietnam/1194/2004 and A/Indonesia/05/2005 (n = 26)
Age group (years) (%)			
20-29	33 (46)^a^	21 (62)	10 (38)
30-44	27 (38)	10 (29)	13 (50)
45-60	11 (15)	3 (9)	3 (12)
Gender, Female (%)	51 (72)	8 (24)	2 (8)

^a^Because the percentage in each age group was rounded off, the sum of the numbers in the column was added up to 99% only.

The common solicited systemic reaction was fatigue (26% and 18% for the first and second dose of immunization, respectively) and general muscle ache (43% and 24% for the first and second dose of immunization, respectively). The rates of solicited systemic reaction were generally higher in participants with previous A/Vietnam/1194/2004 and A/Indonesia/05/2005 priming than those without priming or those with previous A/Indonesia/05/2005 priming. Significant difference in the rates of solicited systemic reactions were shown for headache after first dose of immunization (p = 0.03) and muscle ache after first dose of immunization (p = 0.03). After the first dose of immunization, one participant without priming history reports severe general muscle ache that let the participant leave from work for one day. No participant experienced fever greater than 39.5 degree Celsius.

**Figure 3:**
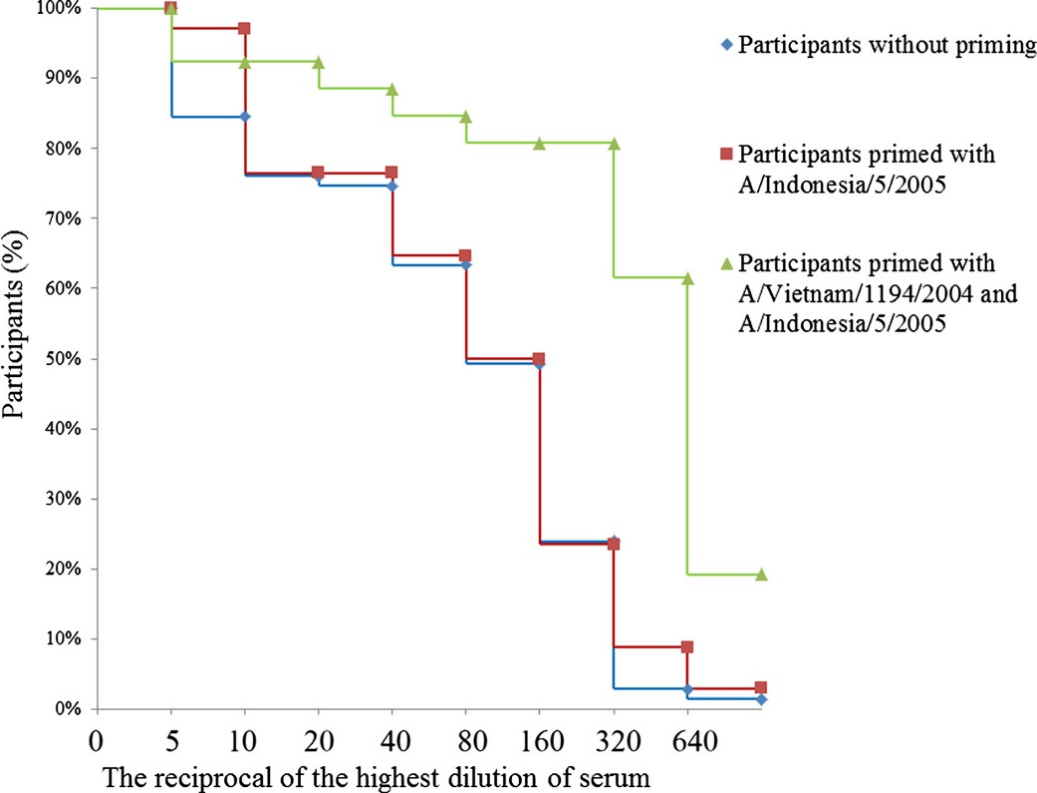
**Reverse cumulative distribution for hemagglutination inhibition titers against A/Vietnam/1194/2004 three weeks after the second dose.**

The HI test results are shown in Table 2. Before the first dose of immunization, 35 (49%), 20 (59%) and 19 (73%) participants were seroprotective in the control group, A/Indonesia/05/2005 primed group and A/Vietnam/1194/2004 and A/Indonesia/05/2005 primed group, respectively. The pre-immunization seroprotective rate and geometric mean titer (GMT) against A/Vietnam/1194/2004 were higher in A/Vietnam/1194/2004 and A/Indonesia/05/2005 primed group than that in the A/Indonesia/05/2005primed group or control group. Three weeks after second dose of immunization, 45 (63%), 23 (68%) and 23 (88%) were seroprotective in control group, A/Indonesia/05/2005 primed group and A/Vietnam/1194/2004 and A/Indonesia/05/2005 primed group, respectively. The seroconversion rate and GMT were also higher in A/Vietnam/1194/2004 and A/Indonesia/05/2005 primed group than those in the A/Indonesia/05/2005primed group or control group. The immunogenicity against A/Vietnam/1194/2004 three weeks after first dose of immunization was similar to that three weeks after second dose of immunization in A/Vietnam/1194/2004 and A/Indonesia/05/2005 primed group (85% vs. 88% in protective rate, respectively; 163 vs. 176 in GMT, respectively). Reverse cumulative distribution curve of the HI response after the second dose of immunization showed similar HI response in the control group and A/Indonesia/05/2005 primed group, but a higher HI response in A/Vietnam/1194/2004 and A/Indonesia/05/2005 primed group (Figure 3).

**Table 2:** **Hemagglutination inhibition (HI) antibody response against the recombinant A/Vietnam/1194/2004 vaccine strain**

	Participants without priming (n = 71)	Participants primed with A/Indonesia/05/2005 (n = 34)	Participants primed with A/Vietnam/1194/2004 and A/Indonesia/05/2005 (n = 26)
**Base line**			
GMT^a^	20 (14–29)	25 (17–39)	73 (40–133)
Seroprotective rate^b^ (%)	49 (37–61)	59 (41–75)	73 (52–88)
**Three weeks after 1** ^**st**^ **immunization**			
GMT	23 (16–34)	32 (20–51)	163 (91–290)
Seroprotective rate (%)	55 (43–67)	65 (46–80)	85 (65–96)
Seroconversion rate^c^ (%)	8 (3–17)	9 (2–24)	27 (12–48)
**Three weeks after 2** ^**nd**^ **immunization**			
GMT	29 (19–45)	41 (24–68)	176 (100–312)
Seroprotective rate (%)	63 (51–75)	68 (49–83)	88 (70–98)
Seroconversion rate (%)	21 (12–32)	21 (9–38)	35 (17–56)

^a^GMT = geometric mean titer. A GMT of HI test result of a dilution of greater than 1:640 was recorded as a HI titer of 640.

^b^Seroprotective rate is defined as the proportion of participants with a HI titer of 40 or greater.

^c^Seroconversion rate is defined as the percentage of participants who have a HI titer of less than 10 before vaccination and a titer of more than 40 after vaccination or have a HI titer of more than 10 before vaccination and at least a four-fold increase in titer after vaccination.

## Discussion

In the report, we describe the safety and the immunogenicity profile of an influenza A/Vietnam/1194/2004 vaccine in recipients without priming or with homologous or heterologous H5N1 priming. To our knowledge, this is the first report on H5N1 vaccine safety and immunogenicity in individuals primed with H5N1 vaccines produced by manufacturers different from the study vaccine. Previous studies have demonstrated the booster effects of H5N1 vaccines produced by the same manufacturers [10],[11],[20]. However, the vaccine available in the pandemic period might be different from those used in the inter-pandemic period in respect of the virus strain, formula, manufacture method and adjuvant system. Our report describes the real scenario of administrating influenza A H5N1 in individuals with risk of influenza A H5N1 exposure.

Generally speaking, presence of local or systemic elicited reactions was higher in participants primed with A/Vietnam/1194/2004 and A/Indonesia/05/2005 than those in other groups. The safety profile of the study vaccine is consistent with that in Banzhoff’s report which described injection site pain and myalgia as the most common local and systemic reactions, respectively [11]. In our report, no serious adverse events were reported. No participant dropped out of the study because of severe post immunization reaction. Although few participants left from work for local or systemic reaction, all the reaction recovered within one week of immunization. Our results show immunization of the study vaccine is well tolerated in healthy individuals regardless of previous H5N1 priming history.

It is somewhat beyond expectation that the immunogenicity of participants primed with A/Indonesia/05/2005 vaccine was similar with that of participants without priming three weeks after second immunization. Risi et al. reported individuals primed with AS03 adjuvanted A/Indonesia/05/2005 vaccine showed good immune response against A/Vietnam/1194/2004 strain after boosting with A/turkey/Turkey/1/2005 (clade 2.2) vaccine [21]; nevertheless, human data on the immunogenicity of participants primed with A/Indonesia/05/2005 vaccine and boosted with A/Vietnam/1194/2004 or other clade 1 vaccines remains limited. Previous animal model showed diverse results in the prime (clade 2.1)-boost (clade 1) immune response. Ikeno et al. reported priming with A/Indonesia/05/2005 vaccine and boosting with A/Vietnam/1194/2004 resulted in low titers of HI antibody against A/Vietnam/1194/2004 in mice model, even after two booster immunization, and suggested antibody-mediated inhibition of naïve B cells is one of the mechanisms resulted in the phenomenon [22]. Sabarth et al. found priming with Vero cell-derived whole virus A/Indonesia/05/2005 based on wild-type strains, followed with A/Vietnam/1194/2004 boosting, could elicit robust immunity against A/Vietnam/1194/2004 in mice model [23]. The true mechanism for the phenomenon in our report remains to be elucidated. In addition to the interaction of prime-boost on immune response, another speculation for the phenomenon is the inadequate immunogenicity of the priming immunization with the whole virus, non-adjuvanted A/Indonesia/05/2005 vaccine. Although previous study has demonstrated that two dose of the A/Indonesia/05/2005 vaccine of either 3.75 μg or 7.5 μg elicited adequate immune response against homologous A/Indonesia/05/2005 strain and heterologous clade 1 A/Vietnam/1203/2004 strain, the immune response after priming with the A/Indonesia/05/2005 vaccine in 2010 was not evaluated and might be inadequate to prime the participants in our study [17].

Our report shows consistent results with previous studies which demonstrated robust immune response in individuals primed with AS03-adjuvanted A/Vietnam/1194/2004 vaccine and boosted by homologous or heterologous immunizaiton [20],[24],[25]. Our report further indicates that participants primed with AS03-adjuvanted A/Vietnam/1194/2004 vaccine showed high immune response after homologous vaccine boosting with different adjuvant system, apart by a long period of 39 months between the priming and the boosting immunization.

A high proportion of the participants showed immunity against influenza A/Vietnam/1194/2004 before immunization. Because human or avian influenza H5N1 infection has not been identified in Taiwan, it is unlikely that the participants obtained the immunity through wild influenza A H5N1 infection [26],[27]. However, this result is different from most of previous studies in European countries that only 1-3% of naïve individuals were immunogenic against influenza A H5N1 virus [15],[28]. Tambyah et al. reported 14% of individuals in Southeast Asia demonstrated pre-existing immunity to A/Indonesia/05/2005 strain before H5N1 immunization [17]. Gioia et al. reported more than 34% of recipients of seasonal influenza vaccine had a rise of neutralization titer >20 fold over baseline to H5N1 virus and suggested that N1 appears to be a target for the cross-type cellular immunity [29]. Seasonal influenza immunization history was not recorded for the participants in the study. However, we have been notified that most of the participants have received two seasonal influenza vaccines during the 2009–10 and 2010–11 influenza seasons and one pandemic influenza H1N1 vaccine in late 2009 in the past two years. Whether the seasonal or pandemic 2009 influenza immunization plays a role in the cross type immunity against the H5N1 virus remains to be elucidated. Further studies are necessary to interpret the cause of the high proportion of immunity against A/Vietnam/1194/2004.

There are limitations in the study. Only HI test was used to test the immunogenicity against A/Vietnam/1194/2004. Stephenson et al. has reported there was marked inter-laboratory variation in HI and neutralization test results, partly attributed to minor protocol differences among laboratories [30]. Although we have carefully performed the HI test according to previous reported method, our results are limited by the inadequate standardization of HI method [18],[30]. Additional tests, e.g., microneutralization test or single radial hemolysis test, may provide auxiliary measurement of the immunogenicity [31],[32]. The participants were enrolled voluntarily without meticulous balance in demographic characteristic distribution among groups. The gender distribution among the groups showed significant difference. Although the HI titers were not confounded by gender (data not shown), the discrepancy of gender distribution among groups might curtail the credibility of the results. The immune response against A/Indonesia/05/2005 vaccine strain might provide additional information on the boost effect of the study vaccine. However, the HI test against A/Indonesia/05/2005 vaccine strain was not performed and the information was not available in the study.

## Conclusion

The influenza A/Vietnam/1194/2004 vaccine adjuvanted with MF59 did not cause severe adverse events. It elicited mostly mild to moderate reactions among participants without priming or with homologous or heterologous priming. After two doses of influenza A/Vietnam/1194/2004 vaccine, 63%, 68% and 88% were in seroprotective status among participants without priming, participants primed with A/Indonesia/05/2005, and participants primed with both A/Indonesia/05/2005 and A/Vietnam/1194/2004, respectively. Participants primed with A/Vietnam/1194/2004 and A/Indonesia/05/2005 showed high immune response after boosting with the first dose of A/Vietnam/1194/2004 vaccine. Compared with control group, participants primed with A/Indonesia/05/2005 vaccine showed similar immunological response after two doses of A/Vietnam/1194/2004 vaccine. The report suggested those with an increased risk of influenza A H5N1 virus exposure may benefit from receiving influenza A H5N1 priming during the inter-pandemic period if the antigenicity of the pandemic influenza strain is similar to that of the priming strain.

## Authors’ contributions

SHW and MTL conceived of the study; carried out the study and interpreted the results. YCT, CHL, CMC, WYW enrolled participants and acquired information and specimens from enrolled participants. YLH carried out the laboratory work. FYC and PC provided the concept of study design; supervised and coordinated the study. All authors contributed to the drafting or revision of the manuscript. All authors read and approved the final manuscript. All authors agree to be accountable for all aspects of the work in ensuring that questions related to the accuracy or integrity of any part of the work are appropriately investigated and resolved.

## Authors’ original submitted files for images

Below are the links to the authors’ original submitted files for images. Authors’ original file for figure 1 Authors’ original file for figure 2 Authors’ original file for figure 3
