# Supplementary figures and images for: The safety and immunogenicity of a MF59-adjuvanted H5N1 prepandemic influenza vaccine in healthy adults primed with homologous or heterologous H5N1 vaccines: an observational study
Source: BMC Infect Dis. 2014 Nov 14;14:587. doi: 10.1186/s12879-014-0587-z (PMC4236496; doi:10.1186/s12879-014-0587-z)

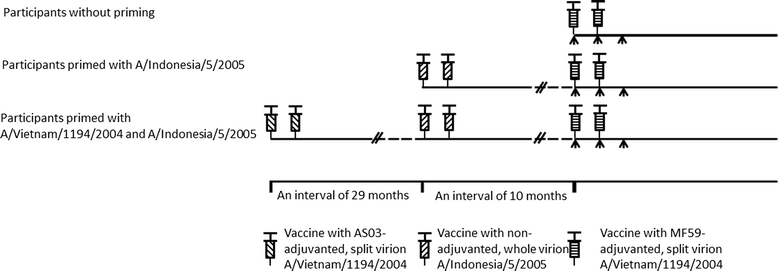

Supplement: Supplementary file 1 — Authors’ original file for figure 1 [file 12879_2014_587_MOESM1_ESM.gif]

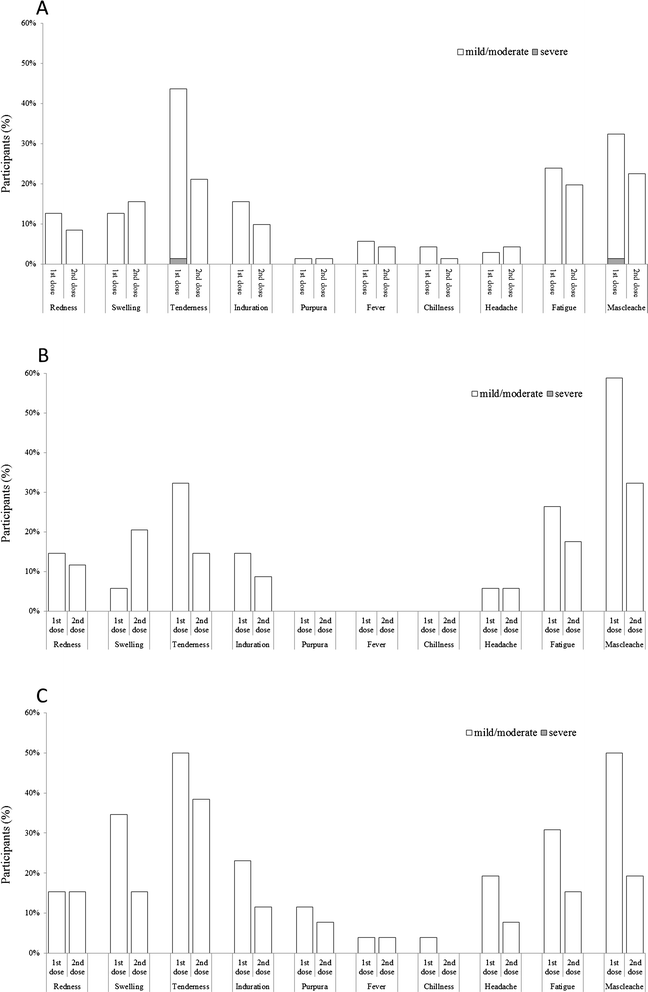

Supplement: Supplementary file 2 — Authors’ original file for figure 2 [file 12879_2014_587_MOESM2_ESM.gif]

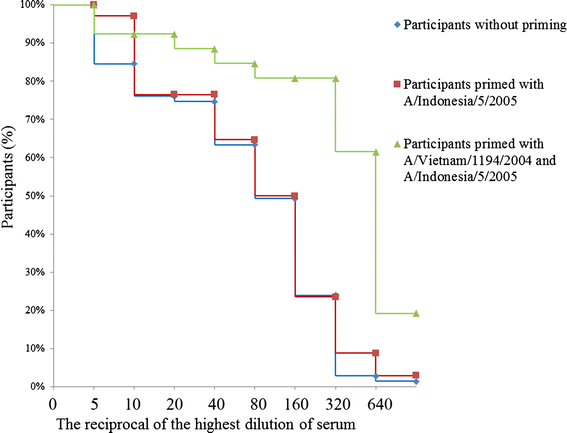

Supplement: Supplementary file 3 — Authors’ original file for figure 3 [file 12879_2014_587_MOESM3_ESM.gif]
